# Viewing Bacterial Colonization through the Lens of Systems Biology

**DOI:** 10.1128/msystems.01383-21

**Published:** 2022-03-31

**Authors:** Madeline R. Barron, Vincent B. Young

**Affiliations:** a Department of Microbiology & Immunology, University of Michigan Medical School, Ann Arbor, Michigan, USA; b Department of Internal Medicine, Division of Infectious Diseases, University of Michigan Medical School, Ann Arbor, Michigan, USA; University of Massachusetts Medical School

**Keywords:** gut microbiota, colonization resistance, systems biology

## Abstract

The gastrointestinal ecosystem is formed from interactions between the host, indigenous gut microbiota, and external world. When colonizing the gut, bacteria must overcome barriers imposed by the intestinal environment, such as host immune responses and microbiota-mediated nutrient limitation. Thus, understanding bacterial colonization requires determining how the gut landscape interacts with microbes attempting to establish within the ecosystem. However, the complicated network of interactions between elements of the intestinal environment makes it challenging to uncover emergent properties of the system using only reductionist methods. A systems biology approach, which aims to investigate complex systems by examining the behavior and relationships of all elements of the system, may afford a more holistic perspective of the colonization process. Here, we examine the confluence between the gut landscape and bacterial colonization through the lens of systems biology. We offer an overview of the conceptual and methodological underpinnings of systems biology, followed by a discussion of key elements of the gut ecosystem as they pertain to bacterial establishment and growth. We conclude by reintegrating these elements to guide future comprehensive investigations of the ecosystem in the context of bacterial intestinal colonization.

## INTRODUCTION

**FIG 1:**
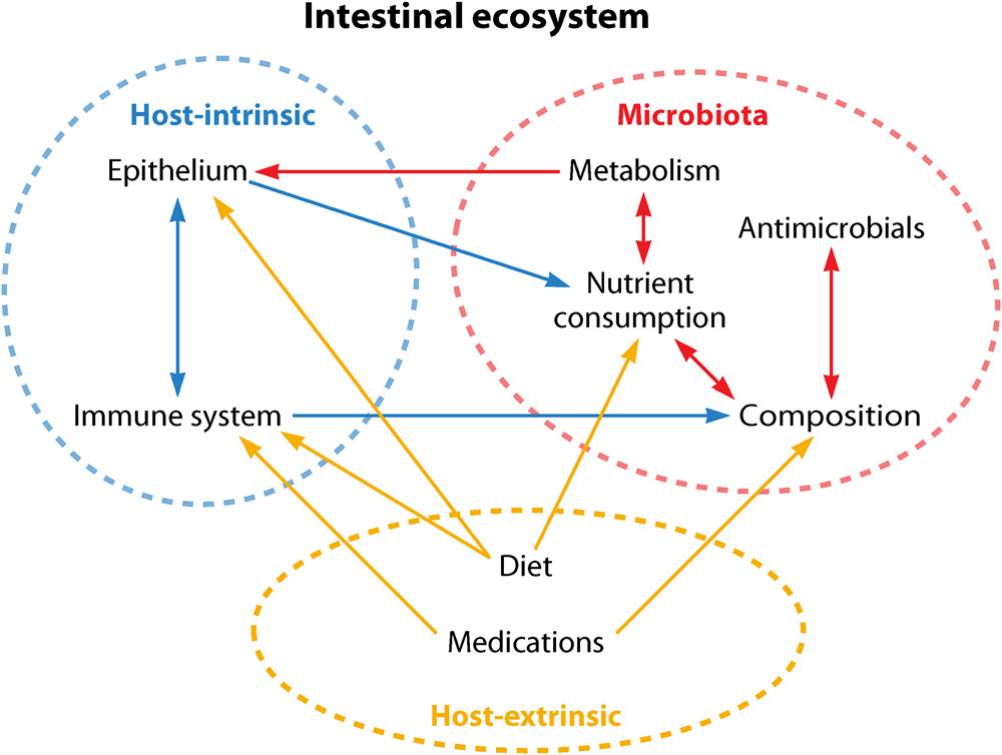
The gut is a complex ecosystem. Factors inherent to the host, microbiota, and external environment interact with others within and between sectors to shape the dynamic intestinal landscape. While there are numerous elements that influence the gut ecosystem beyond those depicted here (e.g., age, pH, oxygen concentrations, lifestyle factors, etc.), we have chosen to highlight only those discussed in this review.

The mammalian gastrointestinal tract is a dynamic ecosystem shaped by interactions between the host, indigenous gut microbiota (the community of microorganisms inhabiting the gut), and external world (Fig. 1). Diverse bacteria continuously attempt to integrate within this ecosystem; while some are harmless and potentially beneficial, others pose a threat to host health. However, regardless of their effects on host well-being, to successfully colonize the gut bacteria must overcome challenges imposed by the intestinal environment, such as host-derived antimicrobial defenses and microbiota-mediated nutrient competition. Understanding bacterial intestinal colonization, therefore, requires uncovering mechanisms by which the gut ecosystem interfaces with microbes attempting to associate with the established community.

The myriad interactions between elements of the gastrointestinal ecosystem make it difficult to detect emergent properties of the system when studied via reductive methods. As such, a systems biology approach, which broadly seeks to understand complex biological systems by studying their components collectively (1), may be the best way to obtain an integrated perspective on the colonization process. However, the use of holistic experimental frameworks for deciphering the complexity of the gut is still in its infancy.

In this minireview, we explore the relationship between the intestinal environment and bacterial colonization from a systems biology standpoint. We first provide an overview of the theoretical and methodological foundations of systems biology. Then, we “break apart” the gut ecosystem to discuss key elements as they relate to bacterial intestinal colonization. Finally, we recombine these elements to highlight the complexity of the gut landscape and provide a basis for future integrative investigations of this landscape as it pertains to bacterial establishment and growth.

## SYSTEMS BIOLOGY: AN OVERVIEW

Systems biology seeks to investigate complex systems, from the inner workings of a single cell to the multifaceted gut landscape, by examining the behavior and relationships of all elements of the system (1). Conceptually, the approach is rooted in the theory of emergence, which posits that the characteristics and functions of complex systems are not entirely deducible from their individual components (2, 3). To that end, emergent properties are those that cannot be assigned to single system elements but stem from the “togetherness” of those elements (3). Colonization resistance is a relevant example, whereby the interplay between the host and microbiota and their interactions with the external environment prevent integration of foreign microbes into the gut ecosystem (4, 5). As such, the permissiveness of the gut is a novel attribute of the system that varies depending on the nature of interactions between its composite parts.

Methodologically, systems biology sits at the crux of technology, computation, and biological experimentation (1, 6). “Omics” technologies have been foundational to the field by allowing researchers to broadly profile the genomic, metabolic, transcriptomic, and proteomic facets of systems in response to specific perturbations (e.g., genetic deletions, disease, introduction of a pathogen, etc.) in biological models (6). In the context of the gut, these models can range in complexity, from simple *in vitro* cell culture, through germfree animals, to vertebrates with replete microbial communities, including humans. The choice of model depends on the experimental goals and the type of data to be collected; the degree of model complexity depends on the components of the system to be analyzed (6). Thus, it can be beneficial to use models of various complexities to gain diverse insights into the functions of system elements.

Once generated via omics methods, high-throughput data are integrated using computational modeling to visualize and explore complex phenomena (6). While beyond the scope of this review, there are multiple discussions of the different modeling schemes for systems biology (6–9). Ultimately, constructing and refining a model is an iterative process, one which yields new hypotheses that can be experimentally tested and validated. The results of these experiments produce data that seed new questions to promote model refinement, hypothesis generation, biological experimentation, and subsequent data integration and modeling, thus highlighting the repetitious nature of systems biology (1).

Because of its emphasis on holistic, interactions-based research, systems biology is often viewed in direct opposition to reductionist approaches that have historically dominated biological research (1, 6). However, reductionist methodologies are not distinct from, but part of, holistic experimental frameworks. Indeed, prior to integration of a system, it is necessary to define its components and characterize the functions and responses of those components by methodically perturbing and monitoring the system, as outlined above (1). These steps lie at the heart of reductionist science, which emphasizes dissecting parts of a system to understand its workings as a whole; systems biology builds on this framework by elucidating relationships between those parts and characterizing properties resulting from their interactions (6, 10). This synergy of holistic and reductive methodologies under the systems biology umbrella is perhaps best summarized by Francis Crick, who stated that “while the whole [system] may not be the sum of the separate parts, its behavior can, at least in principle, be *understood* from the nature and behavior of its parts *plus* the knowledge of how all those parts interact” (11).

## “BREAKING APART” THE GUT ECOSYSTEM

As discussed, systems biology is defined by iterative cycles of experimentation and modeling. The approach involves delineating a system’s components and then combining those components to describe the system at large. To the first point, a great deal of work has been done to understand specific gut environmental factors derived from the host, microbiota, and external world that modulate bacterial intestinal colonization. We highlight some of these factors below, focusing on those that are well established and backed by strong experimental evidence. Such knowledge provides a basis for holistic investigations of the ecosystem moving forward.

## HOST-INTRINSIC FACTORS

The host is traditionally viewed as providing the structural and biochemical bases of the intestinal ecosystem; the epithelium and immune system are two such host-intrinsic pillars of the gut environment (Fig. 1). These factors directly and indirectly interface with microbes to promote or inhibit their growth. Essentially, they serve as filters that actively select for the integration of certain microbes into the gut ecosystem while excluding others.

### The intestinal epithelium.

The intestinal epithelium is a single layer of diverse cell types lining the gastrointestinal tract that exhibit unique functions, such as mucus secretion and production of antimicrobial compound or hormones (12). The epithelium forms a dynamic barrier between the lumen and host circulation that allows selective passage of macromolecules across the intestinal wall (13). Epithelial secretion of antimicrobials, including antimicrobial peptides like defensins and cathelicidins, proteins like lysozyme and calprotectin, and C-type lectins such as RegIII-γ, prevent bacteria from traversing this barrier (14, 15). Moreover, these compounds influence which bacteria will survive and thrive within the gut (16, 17). For example, epithelial-derived antimicrobials inhibit intestinal colonization and survival of Salmonella enterica serovar Typhimurium and Listeria monocytogenes (18, 19). Lysozyme deficiency in mice promotes expansion of lysozyme-sensitive, mucus-degrading bacteria, such as Ruminococcus gnavus and Akkermansia muciniphila (20), indicating that lysozyme contributes to the host barrier against these species. Similarly, RegIII-γ-deficient mice have more bacteria directly associated with their small intestinal epithelium than wild-type animals, demonstrating that RegIII-γ promotes physical separation between luminal bacteria and the epithelial surface (15).

The epithelium also offers spatial and nutritional niches that support microbial growth. For instance, mucus secreted by the epithelium forms a scaffold for bacterial adherence (21), provides nutrients for microbial consumption, contributes to spatial organization of bacterial communities (e.g., by impacting transport of nutrients throughout the gut), and provides regulatory signals that influence bacterial behavior and survival within the gut landscape, such as biofilm formation (22). Thus, the epithelium serves as a framework on which bacterial populations are built and regulated.

### The immune system.

Innate and adaptive immune cells reside below the epithelial surface in the lamina propria, as well as in Peyer’s patches and mesenteric lymph nodes embedded within the epithelium, where they sample and engage with intestinal bacteria (13, 23). Host secretion of cytokines, antibodies, and antimicrobial compounds determines how bacteriologically hostile the gut is, and which microbes are targeted, at a given time (24). For example, immunoglobulin A (IgA) secreted by plasma cells is abundant at mucosal surfaces and is integral in regulating the composition of gut bacterial populations (25). IgA can bind bacteria to limit motility and invasion, as well as aggregate microbes to promote their elimination from the gut (25, 26). On the other hand, IgA can promote colonization of bacteria, such as Bacteroides fragilis, by helping them anchor to the epithelial surface (27). Whether the immune system tolerates or inhibits bacteria depends on the microbe, and how the host distinguishes between friend and foe is not entirely understood. Indeed, a misguided immune attack on symbiotic organisms is a well-recognized feature of chronic intestinal diseases like inflammatory bowel disease (28).

Beyond direct interactions between bacteria and immune cells or their secretory products, immune responses alter the nutritional landscape of the gut to make it hospitable or hostile to specific microbes. For example, pathogens like *S.* Typhimurium, Vibrio cholerae, and Clostridioides difficile capitalize on nutrients liberated from host cells during infection-associated inflammation to occupy a niche that is absent under homeostatic conditions (29–32). However, intestinal inflammation can also limit concentrations of bacterially coveted micronutrients, such as zinc, thereby preventing colonization and infection (33). In addition to metabolic modifications, inflammation increases intestinal oxygen concentrations, which promote expansion of aerobic *Enterobacteriaceae* species while inhibiting growth of anaerobic bacteria (34–36).

## MICROBIOTA-ASSOCIATED FACTORS

The intestinal environment is densely populated, with nearly 100 trillion microbes inhabiting the gut (37). To establish residence in the gut, bacteria must secure sufficient resources to survive within the intestinal ecosystem. The indigenous gut microbiota determines the relative hostility of the intestinal landscape to invading microbes by creating nutritional niches in the intestine, releasing metabolic by-products that facilitate or inhibit growth of other bacteria, producing signaling molecules that foster communication between microbiota members, and secreting compounds that target and kill microbial competitors. As a result, the ability of bacteria to engraft within the gut is largely regulated by the metabolic and antimicrobial defenses of the established bacterial community.

### Microbial nutrient competition, cross-feeding, and production of metabolites.

By sequestering nutrients within the intestine, the indigenous microbiota constitutes a barrier to colonization and growth of adventitious bacteria. The importance of nutrient availability in shaping gut bacterial populations was first recognized by Rolf Freter, whose “nutrient niche” hypothesis posits that populations of gut bacteria are controlled by competition for distinct nutritional niches, and that each particular species is more efficient than others in utilizing one or a few specific substrates (38). The population of a given species, therefore, is regulated by the concentrations of these limiting substrates.

For instance, in one proof-of-concept study, Bacteroides ovatus was engineered to metabolize the marine polysaccharide porphyran, a substrate that is not endogenous to the murine diet, thus equipping this gut bacterium to stably engraft in the gut of porphyrin-fed mice in the presence of a complex microbiota (39). Without porphyran supplementation, the bacteria were excluded from the gut (39). Another study illustrated that three nonpathogenic strains of Escherichia coli collectively eliminated a pathogenic strain (EDL933) from the gut of streptomycin-treated mice, despite exhibiting variable individual success in reducing EDL933 colonization (40). These results suggest that indigenous E. coli strains saturate available niches for invading strains and, in light of Freter’s hypothesis, consume distinct nutrients to coexist in the gut (40, 41). However, such coexistence could also be explained by an alternative theory, known as the Restaurant hypothesis (42, 43), which theorizes that organisms with the same nutritional preferences can coexist within the gut if they reside in spatially distinct biofilms (42, 43). The hypothesis emerged from work postulating that E. coli occupies mixed mucosal biofilms (“restaurants”), where it consumes polysaccharides released by other, namely anaerobic, species (44, 45). The Restaurant hypothesis refines Freter’s theory by adding a spatial dynamic to microbial competition and coexistence within the gut. Both hypotheses, however, highlight the necessity of nutrient accessibility for bacterial intestinal colonization and growth.

In addition to nutrient utilization and competition, the microbiota produces thousands of metabolites that modulate intestinal microbial community composition and behavior. For example, short-chain fatty acids (SCFAs) generated via microbial fermentation of dietary fiber serve as a food source for microbes and support cross-feeding relationships (46–48). SCFAs can also be directly toxic to bacteria by dissociating within and acidifying the intracellular environment (49). As such, SCFAs prevent potentially pathogenic bacteria from colonizing the gut (50). Indeed, butyrate can inhibit growth of C. difficile, and a reduction in SCFA levels in murine models of C. difficile infection is associated with an altered microbiota and susceptibility to infection (51–53).

Bile acids represent another class of microbiota-associated metabolites with well-recognized roles in bacterial intestinal colonization. These compounds disrupt bacterial membrane integrity and induce DNA damage and oxidative stress, among other inhibitory effects (54). Primary bile acids are produced in the liver and conjugated to the amino acids taurine and glycine (54); upon passage into the large intestine, they are metabolized by members of the microbiota via deconjugation and 7α-dehydroxylation. The resulting secondary bile acids are particularly important for inhibiting growth of a range of bacteria, including members of the genera *Lactobacillus* and *Bifidobacteria* (55) and C. difficile (56, 57).

In addition to these inhibitory compounds, the microbiota also secretes signaling molecules (i.e., autoinducers) that, via quorum sensing, regulate the density and behavior of intestinal bacterial populations. For instance, Thompson and colleagues demonstrated that treatment with streptomycin alters the microbiota of mice, leading to an enrichment in members of the phylum *Bacteroidetes* and a decrease in *Firmicutes* species (58). However, when antibiotic-treated mice were colonized by a strain of E. coli capable of producing high levels of AI-2, an autoinducer that fosters cross-species communication, animals exhibited an increased abundance of *Firmicutes* and fewer *Bacteroidetes* species (58). This study points to bacterial communication as an important regulator of microbial cooperation and community structure within the gut ecosystem.

### Microbiota production of antimicrobial compounds.

In addition to the aforementioned metabolites, bacteria secrete antimicrobial compounds that directly target and kill other bacteria, thus giving the secreting cell a competitive edge (59, 60). For example, bacterial cells produce peptides called bacteriocins, which come in various sizes and structures, and elicit their bactericidal activity in several ways, including forming pores in target cell membranes and inhibiting DNA, RNA, or protein synthesis (61–66). Bacteriocins are highly prevalent within the microbiota and are secreted by both Gram-positive and Gram-negative bacteria; lactic acid bacteria, including members of the genera *Lactobacillus* and *Enterococcus*, are some well-known producers (67). Importantly, bacteriocins can promote resistance to colonization by enteric invaders, like Yersinia enterocolitica, Salmonella enterica, and L. monocytogenes (60, 68, 69).

While some antimicrobial compounds are secreted into the extracellular milieu, others are directly injected into competitors via the type VI secretion system (T6SS), a mechanism employed by both pathogenic and nonpathogenic bacteria (69, 70). Effectors secreted by T6SS include cell wall-degrading enzymes, pore-forming toxins, and nucleases, and gut microbes employ T6SS to effectively colonize the gut. For example, B. fragilis uses a T6SS to deploy toxins that antagonize other *Bacteroidales* species and create a niche within the intestine (71).

## HOST-EXTRINSIC FACTORS

The external world plays an important role in regulating conditions within the gut environment and the bacterial populations that reside there. Factors associated with host lifestyle, such as exercise and smoking, affect intestinal processes like gut transit time and gut microbiota composition and metabolism, which impact bacterial intestinal colonization and proliferation (72, 73). The bacteria present within a host’s surroundings determine which species contact the gut in the first place. For example, mode of delivery at birth (e.g., vaginal birth versus Cesarean section) dictates whether “seeding” gut bacterial populations are primarily maternal or environmental (74). Moreover, the composition of the microbiota varies with age, whereby the community rapidly expands and diversifies from birth through early childhood before reaching a relatively stable state characteristic of adulthood (75, 76). However, as is true for host-intrinsic and microbiota facets of the gut environment, there are several extrinsic factors with paramount roles in modulating which bacteria survive and thrive in the gut. Diet and medications are two examples (Fig. 1).

### Diet.

Diet is one of the most important modulators of the intestinal environment; it influences host intestinal physiology and function and controls which bacteria will be “well fed” within the gut. Dietary constituents, such as fiber, protein, fat, vitamins, and sugars, impact host intestinal function in different ways, including by regulating immune responses and the integrity of the epithelial barrier (77). Moreover, diet affects host digestive processes and metabolism. For example, increased bile secretion in mice fed a high-fat diet promotes intestinal colonization by *S.* Typhimurium, which has higher bile resistance than other gut bacterial colonizers (78). Thus, food can select for bacteria that survive in the potentially hostile metabolic landscape of the intestine.

As discussed above, nutrient availability plays a major role in determining the composition of the gut microbiota. Notably, though microbiota composition is largely stable, daily changes in diet can transiently alter community structure (79, 80). Such changes are partially modulated by the community itself; the microbiota regulate host appetite (81) and may influence dietary choices (82, 83) via the gut-brain axis, highlighting the cross talk between host and microbiota in shaping the intestinal nutritional repertoire. Along these lines, gut microbiota structure fluctuates in response to host circadian rhythms and feeding patterns (84, 85), which likely reflects temporal variations in the nutritional landscape of the gut. As such, diet serves to shape the competitive pressures on the microbial community. For instance, low-fiber diets promote expansion of mucus-degrading bacteria and subsequent susceptibility to mucosal pathogens (86). In contrast, high-fiber diets create a nutritionally permissive environment for bacteria that degrade complex carbohydrates, like those of the genus *Bacteroides* (e.g., Bacteroides thetaiotaomicron) (87). Interestingly, a diet high in simple sugars (glucose and sucrose) inhibits B. thetaiotaomicron from colonizing the mouse gut (88). These sugars suppress expression of colonization factors needed for B. thetaiotaomicron to become established and persist within the gut, suggesting that dietary components effect bacterial physiology beyond acting as a food source (88).

### Drugs: antibiotics and beyond.

Medications and other xenobiotics regulate the composition and function of gut microbial populations. Antibiotics can affect the structure of the intestinal bacterial community by obliterating large swaths of the gut microbiota, thus opening niches for bacteria that might normally be barred from the gut. Nonantibiotic medications also influence bacterial survival and proliferation within the intestine. Drugs, including proton pump inhibitors (PPIs; used to decrease stomach acid), metformin (an antidiabetic), laxatives, and antipsychotics, among others, are associated with structural and functional changes in the gut microbial community (89, 90). PPIs in particular are known for increasing risk for infection by enteric pathogens, like C. difficile (50). While the mechanisms are relatively unclear, it is likely that such drugs modulate the gut microbiota in both indirect and direct ways. For instance, PPI-induced reduction in stomach acid may inadvertently select for bacterial species that are normally suppressed (91). Similarly, drugs that target the host immune response, such as biologic therapies and immunosuppressants, influence the inflammatory (and thus antimicrobial) profile of the gut. Indeed, mice administered common immunosuppressants exhibit alterations in gut microbiota structure, as well as decreased expression of antimicrobial peptides within their small intestine (92). These changes are accompanied by an increase in the abundance of endogenous *Enterobacteriaceae* and increased susceptibility to colonization by pathogenic E. coli, suggesting that conditions are favorable for survival of these inflammation-associated microbes (92).

In terms of direct modulation, pantoprazole, a PPI, was recently shown to inhibit the *in vitro* growth of various members of a defined bacterial community isolated from human stool (93). On a broader scale, Maier and colleagues discovered that a range of antipsychotics, antimetabolites, and calcium channel blockers, among other drugs, inhibit the growth of representative gut bacterial colonizers *in vitro* (94). Moreover, therapeutic drugs can bioaccumulate within gut bacterial cells and alter their metabolism, ultimately leading to changes in community composition via formation of new cross-feeding opportunities (95). Together, these findings support the idea that drugs can alter bacterial community composition beyond their host-associated mechanisms of action.

## REINTEGRATING THE GUT ECOSYSTEM

Investigations of host-intrinsic, microbiota, and host-extrinsic factors have yielded essential insight into the elements shaping the structural and functional foundations of the gut ecosystem and their role in bacterial colonization. However, while controlled experimental systems provide the opportunity to study specific aspects of physiology, they miss out on important interactions between system components. Therefore, the challenge in research going forward is how to study specific mechanisms in the context of these complex relationships.

**FIG 2:**
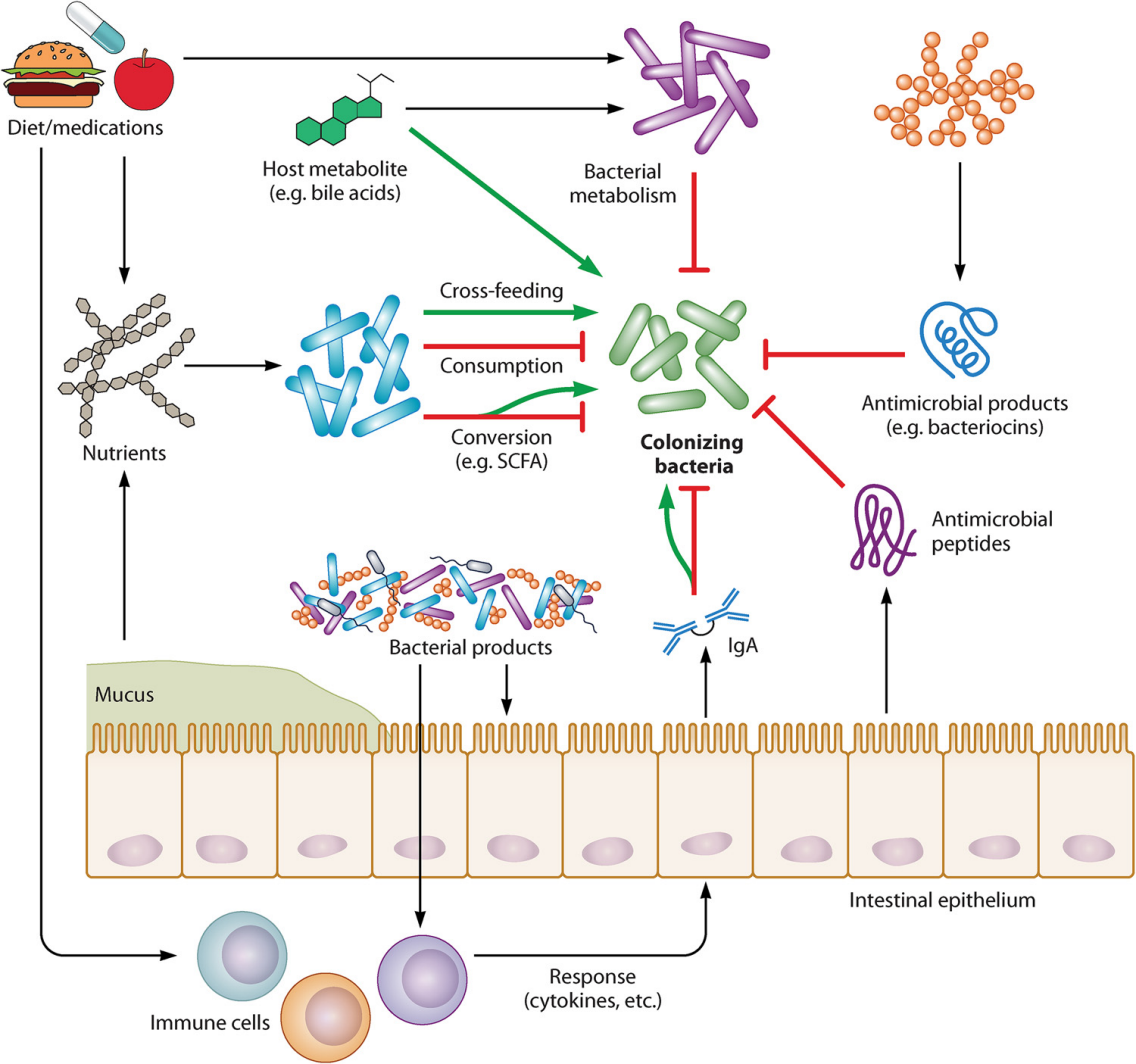
Mechanisms by which the gut ecosystem modulates bacterial intestinal colonization. All facets of the gut landscape are directly or indirectly linked with the others. The gut microbiota influences the success of bacterial colonizers by regulating nutrient availability, producing metabolites that inhibit bacterial growth, and secreting antimicrobial molecules. Microbiota-associated processes depend on host-extrinsic factors (e.g., diet and medications). Moreover, the microbiota shapes the gut landscape via interactions with key host-intrinsic gut environmental factors, like immune cells and the intestinal epithelium, both of which secrete products like antibodies (e.g., IgA) and antimicrobial peptides and mucus, respectively, that further influence the hostility or permissiveness of the gut. The functions of these host-intrinsic elements are also shaped by interactions with one another, as well as other factors, like diet. Green arrows indicate functions that can support colonization, depending on context.

For example, the epithelial barrier continuously interacts with immune cells to modulate their response to intestinal microbes, essentially acting as a portal through which immune-microbe communications are initiated and regulated (96) (Fig. 2). In addition, the gut microbiota regulates epithelial and immune barrier functions, including secretion of mucus, IgA, and antimicrobial peptides, via production of metabolites and other products (97–99) (Fig. 2). The host, in turn, deploys these defenses to shape the composition and metabolic output of the microbiota, which determine the hostility of the metabolic environment for colonizing microbes (Fig. 2). Finally, factors like diet and medications modify host and microbial metabolic and antimicrobial defense mechanisms (97, 98) (Fig. 2); in fact, it is impossible to discuss these factors without accounting for their relationships with the host and microbiota. Furthermore, it is impossible to fully grasp emergent biological phenomena, like colonization resistance, without a holistic view of the gut landscape.

Due to the development of technologies that allow examination of the gut in all its complexity and nuance, investigators have begun adopting a systems biology approach to studying the intestinal ecosystem. Many studies have centered on using integrative methods to mine the microbiome for features, like specific microbial taxa and metabolites, that associate with particular host outcomes, including diseases like IBD, colorectal cancer, obesity, and type 2 diabetes (100–105). There are studies, however, that have employed such methods specifically to investigate bacterial intestinal colonization (106–112). Indeed, by integrating microbiota and metabolomics data with machine learning, researchers predicted microbial and metabolic features associated with susceptibility to intestinal colonization and persistence of C. difficile in antibiotic-treated mice (110). To this end, multiple studies have taken a modeling approach toward identifying bacterial taxa predictive of C. difficile infection (109, 113) and disentangling how indigenous microbes modulate infection by the pathogen (114). Likewise, Midani and colleagues integrated 16S rRNA sequencing data and computational modeling to predict individuals’ susceptibility to V. cholerae colonization after exposure to cholera patients, based on their microbiotas and other clinical and epidemiologic factors (108). Beyond colonization by individual microbes, investigators have also applied systems biological techniques to understand microbial succession in the intestine (112), as well as to determine factors governing successful engraftment of donor bacteria in the gut of fecal microbiota transplant recipients (111).

Nevertheless, while progress has been made, many studies thus far have been partially integrative, methodologically and in terms of how they view the gut landscape. From a methodological standpoint, omics have been extensively employed to profile elements of the gut landscape and their association with bacteria (e.g., the use of microbiota sequencing to identify taxa correlated with intestinal colonization by diverse bacterial species, such as C. difficile, Salmonella enterica, and Lactobacillus reuteri) (115, 116). However, a relatively small number of studies use these data to construct computational models that form the backbone of systems biology. Those that do, including the examples above, tend to be narrow in their scope of the intestinal landscape by focusing on one or a few facets of the environment (e.g., microbiota composition/metabolic output) while leaving others (e.g., host-intrinsic/host-extrinsic elements) out of the equation.

With this in mind, moving toward a comprehensive understanding of gut microbial colonization will require incorporating elements of the gut ecosystem stemming from the host, microbiota, and the external environment into analyses. Technically, it will require adopting a bona fide systems biology framework. This means going beyond omics to computationally integrate high-throughput data and develop models that take all system elements and their interactions into account. These *in silico* efforts will be informed by, and inform, experimentation in intestinal model systems, the number and diversity of which continue to increase as technology advances (e.g., development of organotypic intestinal cell culture, gut-on-a-chip technologies, etc.). Benchmarking biological and computational models will be necessary for delineating their strengths and weaknesses in diverse experimental contexts (105). Moreover, collaborations between bench and data scientists will be key for recognizing and meeting the challenges that come with such complex, multifactorial analyses.

## CONCLUDING REMARKS

Understanding how bacteria integrate within the gut requires thorough, integrative investigations of the intestinal ecosystem. This can be achieved via a systems biology approach informed by new technologies; biological experimentation; advancements in methods for generating, integrating, and analyzing high-throughput data sets; and the increasingly collaborative nature of biological research. Given the demonstrated role of bacteria in host health, there is interest in using bacteria to bolster health and prevent disease. Ultimately, a holistic perspective of the intestinal environment could facilitate the rational design of strategies to promote colonization by symbiotic organisms and prevent colonization by potentially pathogenic ones.
